# Reduction of Decoy Receptor 3 Enhances TRAIL-Mediated Apoptosis in Pancreatic Cancer

**DOI:** 10.1371/journal.pone.0074272

**Published:** 2013-10-25

**Authors:** Wei Wang, Mei Zhang, Weimin Sun, Shanmin Yang, Ying Su, Hengshan Zhang, Chaomei Liu, Xinfeng Li, Ling Lin, Sunghee Kim, Paul Okunieff, Zhenhuan Zhang, Lurong Zhang

**Affiliations:** 1 Department of Radiation Oncology, University of Florida, Gainesville, Florida, United States of America; 2 Department of General Surgery, Second Affiliated Hospital of Fujian Medical University, Quanzhou, Fujian, China; 3 Department of Immunology, Second Military Medical College, Shanghai, China; 4 BioPowerTech, Tuscaloosa, Alabama, United States of America; 5 Central Laboratory, First Affiliated Hospital of Fujian Medical University, Fuzhou, Fujian, China; University of Nebraska Medical Center, United States of America

## Abstract

Most human pancreatic cancer cells are resistant to tumor necrosis factor (TNF)-related apoptosis-inducing ligand (TRAIL)-mediated apoptosis. However, the mechanisms by which pancreatic cancer cells utilize their extracellular molecules to counteract the proapoptotic signaling mediated by the TNF family are largely unknown. In this study, we demonstrate for the first time that DcR3, a secreted decoy receptor that malignant pancreatic cancer cells express at a high level, acts as an extracellular antiapoptotic molecule by binding to TRAIL and counteracting its death-promoting function. The reduction of DcR3 with siRNA unmasked TRAIL and greatly enhanced TRAIL-induced apoptosis. Gemcitabine, a first-line drug for pancreatic cancer, also reduced the level of DcR3. The addition of DcR3 siRNA further enhanced gemcitabine-induced apoptosis. Notably, our *in vivo* study demonstrated that the therapeutic effect of gemcitabine could be enhanced via further reduction of DcR3, suggesting that downregulation of DcR3 in tumor cells could tip the balance of pancreatic cells towards apoptosis and potentially serve as a new strategy for pancreatic cancer therapy.

## Introduction

The balance between proapoptotic and antiapoptotic factors is a major determinant in the fate of tumor cells. These cells manage to tip the balance towards survival by overexpressing antiapoptotic molecules in intracellular and intercellular sites. A body of studies has demonstrated that intracellular B-cell lymphoma 2 (Bcl-2) promotes malignancy in several types of tumors, whereas blocking its function enhances the effect of anticancer treatments [1], [2], [3]. Bcl-2 stabilizes the mitochondrial membrane and prevents the release of cytochrome c from mitochondria and the formation of apoptosomes in the cytoplasm [4], [5], thereby reducing tumor cell apoptosis and enhancing the ability of tumors to grow and metastasize.

However, the majority of apoptotic signaling is activated extracellularly via the binding of proapoptotic ligands from one cell to death receptors on the surface of another cell [6], [7]. For example, the ligands of the tumor necrosis factor (TNF) family bind to their receptors (e.g., TNF to TNFR, FasL to Fas, LIGHT to HVEM/TR2) and then trigger apoptotic signaling in response to unfavorable events [8], [9]. The extracellular mechanism by which cells protect themselves before death ligands bind to their receptors has attracted much attention [10], [11]. Although the discovery of decoy receptors (e.g., DcR1, DcR2, and DcR3) has shed some light on this phenomenon [8], [12], a more detailed understanding of these mechanisms is needed.

Tumor cells utilize 2 layers of protection: (1) an active defense system in which decoy receptors block the death ligand before they reach the receptors of the TNF family on the cell surface and prevent the initiation of death signaling; and (2) a passive defense system in which antiapoptotic machinery plays a role once the death signal is triggered inside the cells. Since portions of the molecules of type I cell-death receptors (e.g., DcR3, DR3, DR5, TNFR1, OPG, and OX40) and death ligands (e.g., FasL, LIGHT, TL1A, TRAIL, and LTA) share functionally similar domains, especially among the 6 members of the TNFR family [13], we used several approaches to explore the binding and interaction of DcR3 with TNF-related apoptosis-inducing ligand (TRAIL). Our results showed that DcR3 binds not only to FasL, TL1A (VEGI), and LIGHT [14], [15], [16], [17] but also to TRAIL, as evidenced by results from several assays, including Biacore binding, flow cytometry, immunoprecipitation, Western blotting, and enzyme-linked immunosorbent assay (ELISA). Malignant pancreatic cancer cells express a high level of DcR3, which can be downregulated by DCR3 siRNA or chemotherapy drugs, such as gemcitabine. The combination of DcR3 siRNA with TRAIL or gemcitabine greatly enhances the apoptotic process *in vitro* and slows tumor growth *in vivo*, suggesting that the downregulation of extracellular antiapoptotic DcR3 may enhance anticancer therapy. This is especially true for aggressive pancreatic cancer, which utilizes DcR3 as the means for its progression.

## Materials and Methods

### Cell lines and reagents

Two human pancreatic cancer cell lines, AsPC-1 and MiaPaCa-2, and a colon carcinoma cell line, SW480, were obtained from the American Type Culture Collection (ATCC, Manassas, VA). Cells were maintained as mono-layer cultures in Dulbecco's modified Eagle's medium (DMEM) supplemented with 10% fetal bovine serum, 100 μg/ml of streptomycin, and 100 U/ml of penicillin at 37°C in a humidified 5% CO_2_ atmosphere. DcR3-related reagents were provided by BioPowerTech (Tuscaloosa, AL). Gemcitabine was purchased from the pharmacy at the University of Rochester Medical Center (Rochester, NY). The siRNA for DcR3 was synthesized at Integrated DNA Technologies, Inc. (Coralville, IA). Recombinant FasL and TRAIL and their antibodies were purchased from R&D Systems (Minneapolis, MN) and BioLegend, Inc. (San Diego, CA). Protein A Sepharose beads were purchased from Pharmacia (Uppsala, Sweden). Anti-cleaved poly (ADP-ribose) polymerase (PARP) polyclonal antibodies were purchased from Cell Signaling Technology, Inc. (Beverly, MA) and anti-glyceraldehyde 3-phosphate dehydrogenase (GAPDH) polyclonal antibody was purchased from Santa Cruz, Inc. (Santa Cruz, CA).

### Biacore assay

The binding of recombinant human TRAIL to human DcR3 was analyzed on a Biacore 3000 instrument (Biacore Inc., Piscataway, New Jersey). DcR3 was covalently conjugated to Biacore sensor flow cells (CM5 chip) via amine groups using N-ethyl-N'-(dimethylaminopropyl) carbodiimide/N-hydroxysuccinimide. Various dilutions of TRAIL (1 to 2048 nM) were passed through the conjugated flow cell and blank surface at 20 µl/minute for a total volume of 70 µl. The amount of bound protein was determined during washing of the flow cell with HBS-EP buffer (10 mM HEPES [pH 7.4], 150 mM NaCl, 3.4 mM EDTA, 0.005% Surfactant P20). To regenerate the flow-cell surface, the bound proteins were washed with 20 mM of NaOH. Recombinant human LIGHT diluted in HBS-EP buffer (1 to 512 nM) was used as a positive control. The association, dissociation, and equilibrium constants were determined with a kinetic evaluation program in the Bia Evaluation software (Biacore, Inc.) using a 1∶1 binding model and the global analysis method.

### Cell-surface binding analysis

AsPC-1 and MiaPaCa-2 cells were incubated with or without recombinant DcR3 (5 μg/ml), TRAIL (5 μg/ml), or murine anti-TRAIL monoclonal antibody (1 μg/ml) in the same or different tubes for 1 hour at 4°C, followed by biotinylated DcR3 monoclonal antibody (0.5 μg/ml), and then fluorescein isothiocyanate (FITC)-conjugated streptavidin secondary antibody for 1 hour at 4°C. After washing, the bound DcR3 on the cell surface was detected with FACStarPlus (BD Inc., Franklin Lakes, NJ). Data were expressed as the percentage of positively stained cells.

### Immunoprecipitation and Western blotting

The physical association of DcR3 with TRAIL was examined with immunoprecipitation (IP) and Western blotting in 3 different settings. To examine the interaction between 2 pure recombination proteins, 100–μl mixture of DcR3 with TRAIL (1 μg/ml) was precipitated with either anti-DcR3 or anti-TRAIL (3–5 μg) with 20 μl of protein A beads slurry. After washing, the bound DcR3-TRAIL complex on the beads was eluted with 30 μl of 1X non-denatured Laemmli sample buffer, electrophoresed on 12% sodium dodecyl sulfate polyacrylamide gel electrophoresis (SDS-PAGE), transferred to a nitrocellulose membrane, detected with biotinylated anti-TRAIL or anti-DcR3 (1–2 μg/ml) followed by streptavidin (SA)-HRP, and visualized with a sensitive chemiluminescent substrate (Pierce, Rockford, IL). To detect soluble DcR3-TRAIL complex in cell culture media and the membrane-bound complex in cell lysate, samples with protease inhibitor cocktail (50 μg/ml of PMSF, 1 μg/ml of aprotinin, leupetin, and pepstatin) were cleaned with protein A beads and then incubated with either anti-DcR3 or anti-TRAIL at 4°C overnight while being gently shaken. The bound DcR3-TRAIL complex on the beads was subjected to Western blotting in a manner similar to that described above.

### ELISA-like binding assay

The binding of DcR3 with TRAIL was examined with a modified ELISA. Wells were coated with recombinant FasL or TRAIL (100 μl of 1 μg /ml). After the plates were washed with washing buffer (0.01% Tween 20 in PBS) and blocked with 5% PBS-T (5% Tween 20 in PBS), 100 μl of recombinant DcR3 (1 μg/ml) with or without recombinant TRAIL or FasL (10–20 μg/ml, for competition) was added to each well and incubated at 4°C overnight. After washing, biotinylated-DcR3 antibody (0.2 ng/ml) was added to each well and incubated at 37°C for 1 hour; thereafter, 100 μl of 1∶4000 streptavidin-horseradish peroxidase (SA-HRP) was added and incubated in wells for 45 minutes at 37°C, washed, visualized with 3, 3′, 5, 5′ tetramethyl benzidine (TMB), and read at a wavelength of 450 nm. In reverse, the plates were coated with DcR3 (100 μl of 2 μg/ml) and then incubated with recombinant FasL or TRAIL (100 μl of 1 μg/ml) with or without DcR3 (20–50 μg/ml, for competition), followed by biotinylated anti-FasL or anti-TRAIL (0.5 μg/ml), SA-HRP, TMB and read at A_450_.

### ELISA for DcR3

DcR3 was quantitatively measured, as described previously 17]. Since DcR3 is a secreted protein, 100 μl of culture media was used. Briefly, it was incubated overnight with an ELISA plate coated with anti-DcR3 McAb (2 μg/ml) at 4°C. After miscellaneous proteins were washed off, the bound DcR3 was detected with biotinylated anti-DcR3 McAb and SA-HRP and visualized with TMB substrate. The unknown sample concentration was determined from the standard curve, which was calculated simultaneously.

### Downregulation of DcR3 with Small Interfering RNA (siRNA)

Small interfering RNA sequences for human DcR3 were designed using the BLOCK-iT RNAi Designer (Invitrogen Life Technologies, Carlsbad, CA). The siRNA sequence was 5′- CGCUGCAGCCUCUUGAUGGAGAUGUCC-3′, and the control siRNA sequence was 5′-AGGUAGUUCCAGAACUGCGUGUAGUGG-3′. They were chemically synthesized for transient knockdown of DcR3 in cell culture. In addition, similar sequences were inserted into the pSilencer expression vector for a stable low-DcR3 expresser. Transfection was carried out with Lipofectamine LTX reagent (Invitrogen Life Technologies) and Opti-MEM media, according to the manufacturer's instructions. The cells with transient knockdown of DcR3 were used within 3 days. The stable transfectants were selected in 50 μg/ml of hygromycin B. The surviving cells were pooled to avoid colony variation and use for *in vivo* study.

### Apoptosis assays

Apoptotic cells were detected with 3 assays. Cells were harvested at 24–48 hours and stained with Annexin V/propidium iodide (PI) for apoptotic cells or fixed in 75% alcohol followed by PI staining for the sub-G1 fraction, according to standard protocol from the manufacturers. The results were analyzed by flow cytometry. Western blotting was performed for cleaved PARP. The harvested cells were lysed with 1% NP-40-Tris lysis buffer containing a cocktail of protease inhibitors, and the protein concentration of lysate was determined with the BCA method (Pierce, Rockford, IL). Protein (30 μg) was loaded onto 10% SDS-PAGE, electrophoresed, transferred to a nitrocellulose membrane, incubated with 0.5 μg/ml of rabbit anti-cleaved PARP or anti-GAPDH (as loading control) followed by anti-rabbit-HRP, and visualized with ECL chemiluminescent substrate.

### Animal ethics

This study was performed in strict accordance with and approved by the University of Rochester (Rochester, NY) Institutional Animal Care and Use Committee (IACUC), the University Committee on Animal Resources (UCAR), as outlined in the *Manual on the Responsible Care and Use of Laboratory Animals*. All efforts were made to minimize suffering and limit animal numbers. Any animals that showed signs of weakness and dehydration due to treatment received a wet food diet. Animals that continued to exhibit signs of weakness were humanely euthanized through cervical dislocation.

### Tumor growth in nude mice

Control transfectants or transfectants with low DcR3 expression (2×10^6^ in 0.2 ml saline) were injected subcutaneously into the hind legs of athymic nude mice (5–6 weeks old) with a 27.5-gauge needle. Tumors were allowed to grow for 7 days (approximately 0.1 cm^3^) before treatment. Mice bearing tumors formed by either control transfectants or transfectants with low DcR3 expression were randomly divided into 2 groups (8 mice/group), treated with saline as a control or gemcitabine (IV 100 mg/kg) twice a week for 4 weeks. For the tumor growth curve, tumor size was measured twice weekly with vernier calipers. The tumor volumes were calculated according to the formula *(L x W2)/2* by measuring tumor length (L) and width (W). At the end of experiment, the tumors were removed and weighed. An ELISA was used to assess the DcR3 level in tumor homogenate (30 μg). Western blotting was performed for DcR3, TRAIL, and cleaved PARP.

### Statistical analyses

All data were expressed as the mean ± standard deviation (SD) of at least 3 determinations, unless otherwise stated. The differences among or between the control and treatment groups were determined by Student's *t-*test or analysis of variance (ANOVA). *P*<0.05 indicated statistical significance.

## Results

### DcR3 bound to TRAIL

TRAIL is a membrane-bound death ligand with low levels under normal culture conditions, and DcR3 is a secreted decoy receptor. Although FasL, TL1A, and LIGHT have been identified as ligands of DcR3 [14], [15], [16], [17], [18], the interaction of DcR3 with TRAIL has not been studied in great detail. Because TRAIL shares functionally similar domains with FasL, TL1A, and LIGHT, we speculated that DcR3, which binds to FasL, TL1A, and LIGHT, might also bind to TRAIL.

**Figure 1:**
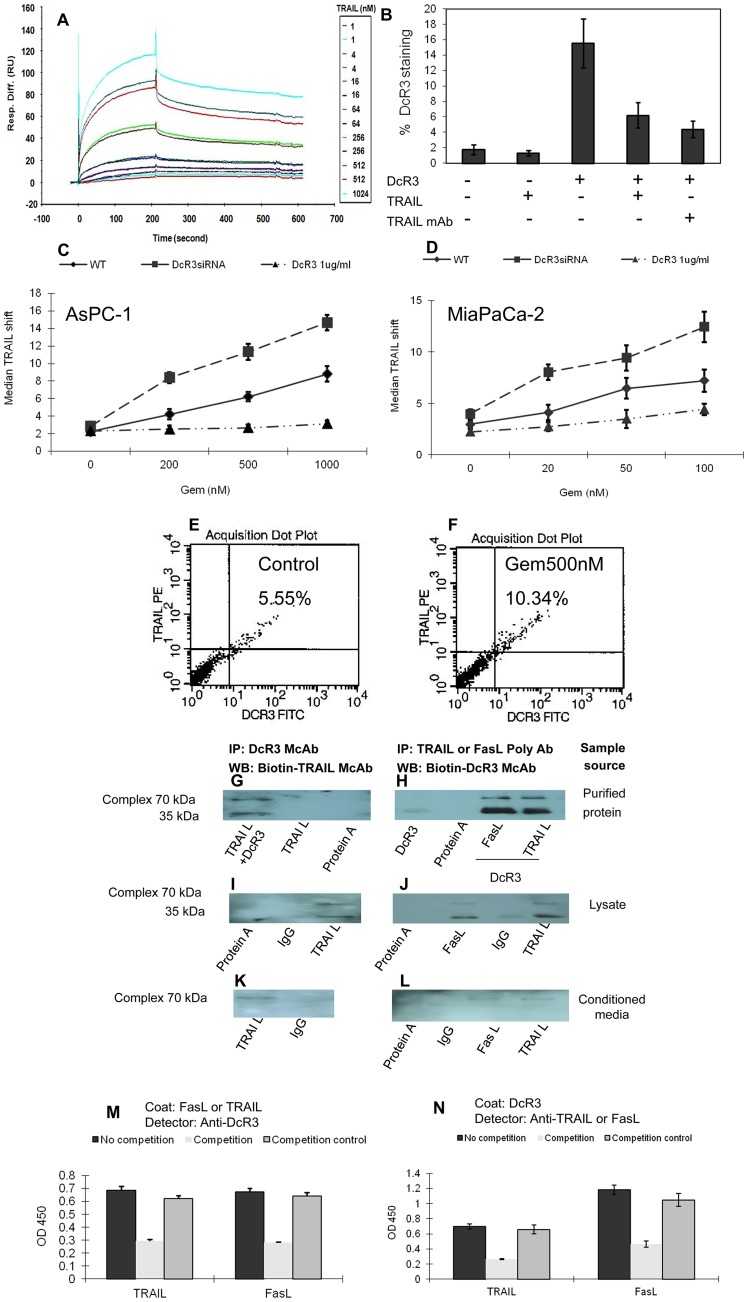
Interaction of DcR3 with TRAIL. Biocore assays, flow cytometry, Western blot, and ELISA-like binding assays were performed to determine the physical interaction between DcR3 and TRAIL. (**A**) **Biacore analysis.** The binding curve of TRAIL to DcR3 was determined at different concentrations (1–2048 nM). The control *K_eq_* of recombinant human LIGHT to DcR3 was 13.2 nM, while the *K_eq_* of TRAIL to DcR3 was 52.8 nM. (**B**) **Flow cytometry for DcR3 with TRAIL on the cell surface.** Suspended AsPC-1 cells were incubated first with PBS, TRAIL (5 μg/ml) without (lane 2) or with DcR3 (lane 4), recombinant DcR3 (lane 3), or mouse anti-TRAIL MAb (1 μg/ml) with DcR3 (lane 5). Thereafter, 5 μg/ml of biotinylated anti-DcR3 was added to determine the binding of DcR3 on cell surface with flow cytometry. (**C and D**) **Flow cytometry for exposed TRAIL.** Gemcitabine was added to AsPC-1 (0–1000 nM) or MiaPaCa-2 (0–100 nM) cells to stimulate the expression of TRAIL. Cells were also treated with PBS, 10 nM of DcR3 siRNA (to reduce the binding and masking of TRAIL), or additional recombinant DcR3 (to block the accessing of anti-TRAIL) to detect TRAIL by flow cytometry. (**E and F**) **Colocalization of DcR3 and TRAIL.** AsPC-1 cells treated without (as control) or with 500 nM of gemcitabine (to enhance the expression of TRAIL) were stained with anti-TRAIL-PE and anti-DcR3-FITC. Doublestained cells were assessed with flow cytometry. (**G to L**) **Immunoprecipitation and Western blotting for DcR3-TRAIL complex.** 70-kDa DcR3-TRAIL complex in 100 μl of pure DcR3 and TRAIL mixture (**G** and **H**), lysate (**I** and **J**), or culture media (**K** and **L**) of AsPC-1 cells was captured with McAb anti-DcR3 (**G, I, K**) or anti-TRAIL (**H, J, L**) McAb and immobilized with 30 μl of protein A beads. The complex of McAb-DcR3-TRAIL was eluted with Laemmli buffer and subjected to Western blotting with biotinylated anti-TRAIL (**G,** **I, K**) or anti-DcR3 (**H, J, L**) followed by SA-HRP and ECL detector. (**M and N**) **Binding competition between free and immobilized forms of DcR3 and TRAIL.** **M**: After 100 μl of 1 μg/ml recombinant TRAIL or FasL was immobilized on an ELISA plate, 1 μg/ml of DcR3 was added without (no competition group) or with 10 μg/ml of TRAIL or FasL (competition group) or boiled TRAIL or FasL (no function protein control), followed by biotinylated anti-DcR3 and SA-HRP and TMB substrate. **N**: DcR3 was immobilized on an ELISA plate. TRAIL and FasL were used as ligands without or with boiled or functional 10 μg/ml of TRAIL or FasL in the presence of DcR3, followed by biotinylated anti-TRAIL or anti-FasL, and SA-HRP and TMB substrate.

To determine whether DcR3 binds to TRAIL, we conducted state of the art Biacore binding analysis. The recombinant pure DcR3 was covalently linked on the flow chip surface, and the pure recombinant TRAIL and LIGHT (as a positive control) were flushed through the surface at various concentrations. **Figure 1** shows the binding curve of TRAIL to DcR3 at different concentrations (1–2048 nM). As shown in **Table 1**, although the control equilibrium constant (*K_eq_*) of recombinant human LIGHT to DcR3 was 13.2 nM, the association rate constant (*K_a_*) and disassociation rate constant (*K_d_*) for recombinant human TRAIL to DcR3 were 1.97×10^4^ 1/Ms and 1.04×10^−3^ 1/s, respectively, and the *K_eq_* was 52.8 nM, indicating binding between TRAIL and DcR3; however, the affinity was lower than that of LIGHT to DcR3.

**Table 1:** Biacore analysis for TRAIL or LIGHT binding to Recombinant DcR3.

Recombinant Protein	*Ka* (1/Ms)	*Kd* (1/s)	*Keq* (nM)
TRAIL	1.94×10^4^	1.04×10^−3^	52.8
LIGHT	4.15×10^4^	5.47×10^−3^	13.2

*K_a_*, association rate constant; *K_d_*, dissociation rate constant; *K* ***_eq_***, equilibrium constant.

To determine whether DcR3 binds to TRAIL, we preincubated AsPC-1 human pancreatic cancer cells with or without recombinant DcR3, TRAIL, or anti-TRAIL McAb, and then stained them with biotinylated anti-DcR3 followed by streptavidin-FITC. Flow cytometry (**Fig 1B**) demonstrated that, while biotinylated anti-DcR3 and streptavidin-FITC alone result in little staining of DcR3, recombinant DcR3 could bind to the cell surface and be detected with anti-DcR3. Due to the competition between free TRAIL and membrane-bound TRAIL, recombinant TRAIL reduced DcR3 cell-surface binding. This binding was also reduced by preincubation with anti-TRAIL, which blocked the accessibility of DcR3 to membrane-bound TRAIL. Together, the data indicate that recombinant DcR3 binds to TRAIL on the surface of AsPC-1 cells.

To better observe the interaction between TRAIL and DcR3, we used gemcitabine, a first-line antipancreatic cancer drug and known TRAIL inducer, to upregulate TRAIL. Due to drug sensitivity differences, AsPC-1 cells (**Fig 1C**) required a 10-fold higher level of gemcitabine to increase the expression of TRAIL, as compared to MiaPACa-2 cells (**Fig 1D**). We hypothesized that soluble DcR3 would mask TRAIL on the cell surface; thus, the reduction of DcR3 would reduce the masking and enhance the staining of exposed TRAIL. To test this hypothesis, we utilized the DcR3 siRNA approach. The reduction of DcR3 reduced masking of TRAIL and increased accessibility of anti-TRAIL in AsPC-1 cells (**Fig 1C**), while the control siRNA did not have such an effect. Likewise, we hypothesized that increased DcR3 surrounding the cells would enhance the masking and reduce the accessibility of anti-TRAIL to TRAIL. Recombinant DcR3 was added to the cells, thereby causing reduced TRAIL staining as detected by flow cytometry (**Fig 1C**). A similar pattern was observed in MiaPaCa-2 (**Fig 1D**), suggesting that the phenomenon of masking TRAIL with DcR3 is common in the tested cell line. These results strongly support the notion that DcR3 is associated with TRAIL on the cell surface.

To further determine the binding of DcR3 to TRAIL, we used gemcitabine to trigger the overexpression of TRAIL [19], [20] and added recombinant DcR3. Double staining with anti-TRAIL-PE (red) and anti-DcR3-FITC showed an enhanced colocalization (**Fig 1F**), as compared to the control (**Fig 1E**), suggesting that DcR3 is physically associated with TRAIL.

To confirm the physical association of DcR3 with TRAIL, we performed immunoprecipitation and Western blotting. FasL, a known ligand for DcR3, was used as a positive control. The results showed that the anti-DcR3-captured DcR3-TRAIL complex (approximately 70 kDa MW under mildly denaturing conditions) could be detected by anti-TRAIL in a mixture of pure recombinant DcR3 and TRAIL (**Fig 1G**), lysate (**Fig 1I**), or conditioned media (**Fig 1K**) of AsPC-1 cells that expressed both DcR3 and TRAIL. Likewise, the anti-TRAIL-captured DcR3-TRAIL complex could be detected by anti-DcR3 in the same 3 settings (**Fig 1H, 1J,** and **1L**). As a side-by-side positive control, FasL exhibited a pattern similar to that of the anti-FasL-captured DcR3-FasL complex detected by anti-DcR3 (**Fig 1G, 1I,** and **1K**), which strongly supports the notion that TRAIL and FasL bind to DcR3. Notably, the DcR3-TRAIL complex formed not only in a pure protein condition (**Fig 1G** and **1H**) but also in a natural condition in the membrane-bound form (in lysate, **Fig 1I **and **1J**) and in the secretory form (in media, **Fig 1K **and **1L**). The bands of DcR3-TRAIL complex in media were much finer than the bands in lysate, indicating that the complex was membrane bound. These results were consistent with those obtained through flow cytometry.

To further confirm the association of DcR3 with TRAIL, we performed an ELISA-like assay. When TRAIL or FasL was coated onto ELISA plates, the binding of DcR3 to these ligands could be detected with anti-DcR3 and reduced by the addition of free recombinant TRAIL or FasL, which competed with DcR3 for the immobilized TRAIL or FasL (**Fig 1M**). In contrast, when DcR3 was coated onto ELISA plates, the recombinant TRAIL or FasL bound to immobilized DcR3 and was detected by anti-TRAIL or anti-FasL, which was also partially blocked by the free form of DcR3 (**Fig 1N**).

In all tests, the FasL-DcR3 complex served as a side-by-side positive control and showed a pattern similar to that of the DcR3-TRAIL complex (**Fig 1G, 1I, 1K** **. 1L and 1M**), strongly supporting that TRAIL is indeed a new ligand of DcR3.

### Alteration of DcR3 affected gemcitabine-induced apoptosis

**Figure 2:**
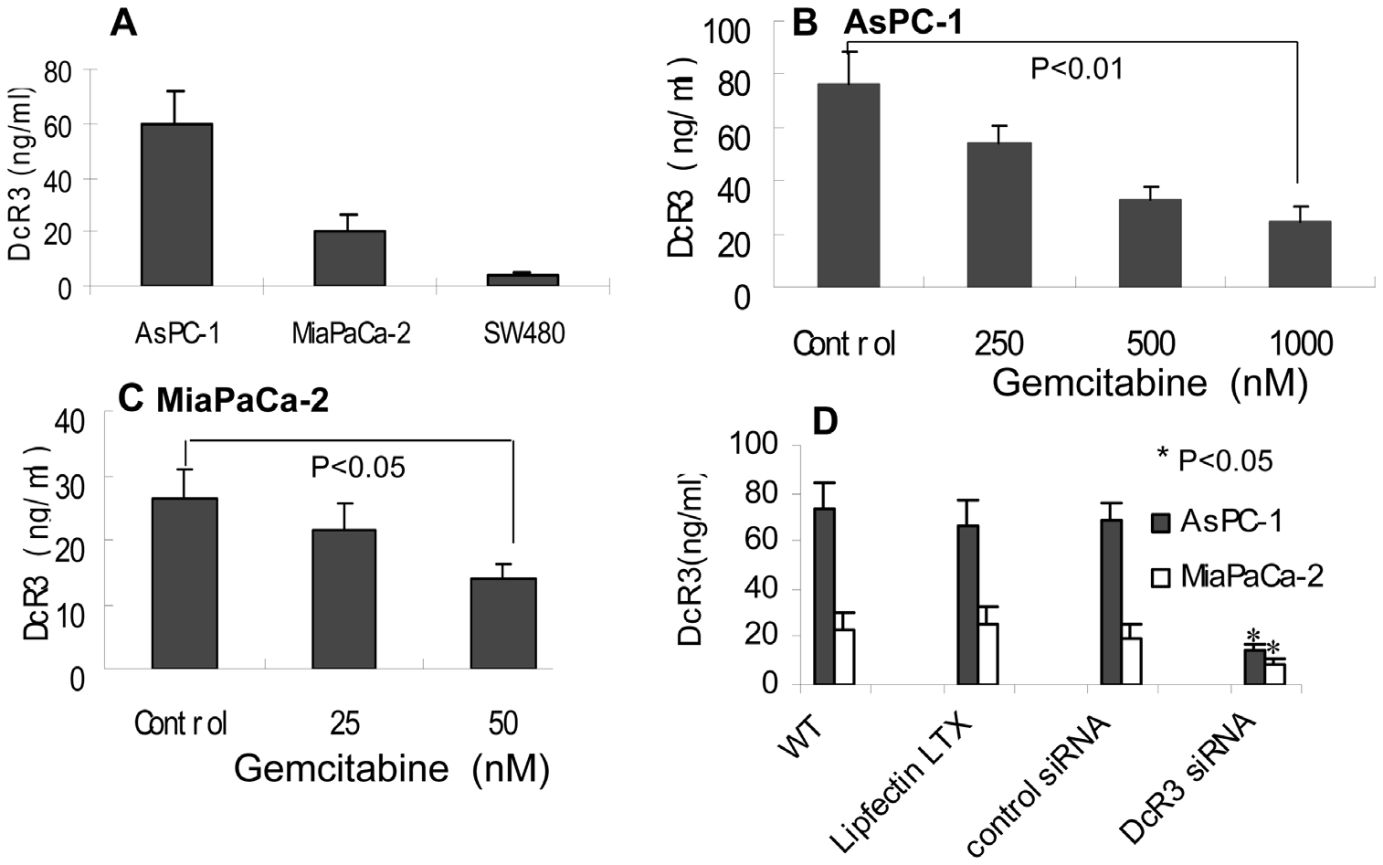
High level of DcR3 in pancreatic cancer cells and downregulation by siRNA and gemcitabine. (A) Expression of DcR3 in AsPC-1, MiaPaCa-2, and SW480 cells (as positive control). The DcR3 level in 100 μl of conditioned media from cells that were cultured in 6-well plates (4 ml) was measured with an ELISA. (B and C) Gemcitabine reduced DcR3 expression. AsPC-1 and MiaPaCa-2 cells were treated with gemcitabine at the indicated concentrations for 48 hours. The DcR3 level was measured in 100 μl of media from each group. (D) siRNA knocked down DcR3 expression. Cells (1×10^5^) in 6-well plates were transfected with 10 nM of DcR3 siRNA or control siRNA in Transfectin LTX (6.25 μl/well). After 24 hours, DcR3 in culture media was measured with an ELISA.

To study the biological function of DcR3 in pancreatic cancer, we measured the level of DcR3 by a quantitative ELISA for DcR3 [21] in AsPC-1 and MiaPaCa-2 pancreatic cancer cells. Compared with SW480, a human colon adenocarcinoma cell line that is known to express high levels of DcR3, as compared to other 13 cell lines [22], the AsPC-1 and MiaPaCa-2 cells had an even higher level of DcR3 (**Fig 2** **A**), which is consistent with other reports [23], [24]. Both DcR3 and TRAIL expressed at a high level on the same cells, suggesting that antiapoptotic and proapoptotic molecules are balanced at a high level to counteract each other. The natural expression of high levels of both molecules makes the study of their interaction much easier.

To determine the interactive effect of DcR3 and TRAIL on cancer cells, gemcitabine and siRNA for DcR3 were used to regulate the level of DcR3. The AsPC-1 and MiaPaCa-2 cells were treated with different doses of gemcitabine for 48 hours, and the level of DcR3 in the media was measured with an ELISA. The results showed that gemcitabine reduced DcR3 in a dose-dependent manner in both cell lines (**Fig 2B** and **C**). A similar reduction was observed with siRNA (**Fig 2D**).

**Figure 3:**
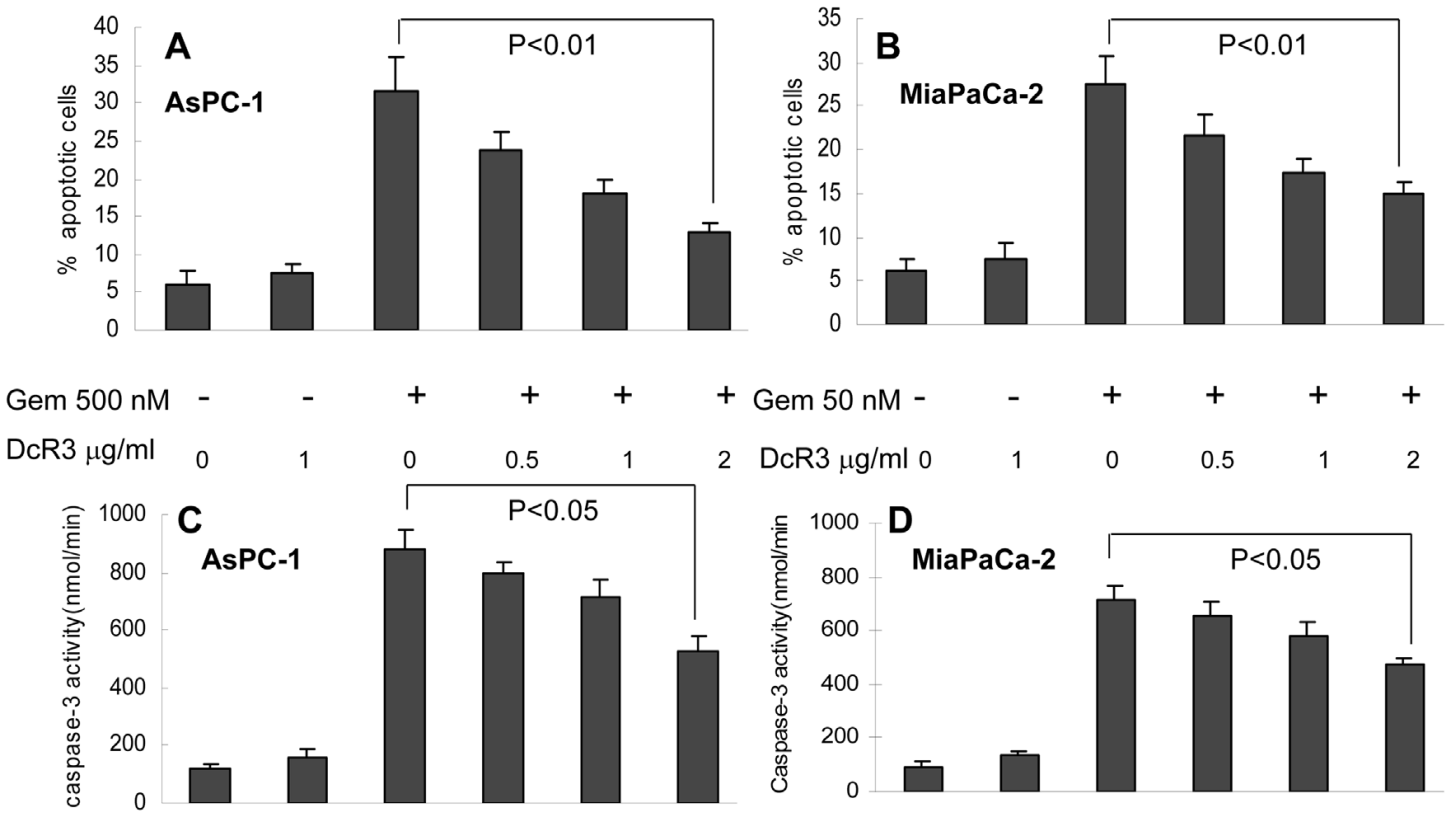
DcR3 reduced gemcitabine-induced apoptosis. Gemcitabine was added to media of AsPC-1 (500 nM) or MiaPaCa-2 cells (50 nM) incubated with 0, 0.5, 1, or 2 μg/ml of recombinant DcR3 for 24 hours. Cells were then subjected to flow cytometry for the sub-G1 fraction (**A** and **B**) or an enzyme assay for caspase 3 activity (**C** and **D**). Gemcitabine-triggered apoptosis was decreased by the addition of DcR3 in a dose-dependent manner (*P*<0.01).

If the function of gemcitabine-reduced DcR3 is to tip the balance towards apoptosis, then the addition of DcR3 should reverse gemcitabine-induced apoptosis. Thus, AsPC-1 and MiaPaCa-2 cells were treated with different doses of gemcitabine with or without recombinant DcR3 for 48 hours, and then the apoptotic cells were measured with Annexin V staining. The results showed that 5–8% of cells were apoptotic in the control, which increased to approximately 30% in the gemcitabine group. This gemcitabine-induced apoptosis was reduced by the addition of recombinant DcR3 (0, 0.5, 1, and 2 μg/ml) in a dose–dependent manner **(** **Fig 3A** and **3B** **)**. Similarly, the gemcitabine-induced increase of caspase 3 activity was reversed by DcR3 (**Fig 3C and 3D**). These results suggest that DcR3 plays a critical role in the inhibition of apoptosis and that the gemcitabine-induced reduction of DcR3 might be related to its antitumor activity.

### Downregulation of DcR3 with siRNA promoted TRAIL-mediated apoptosis in vitro

**Figure 4:**
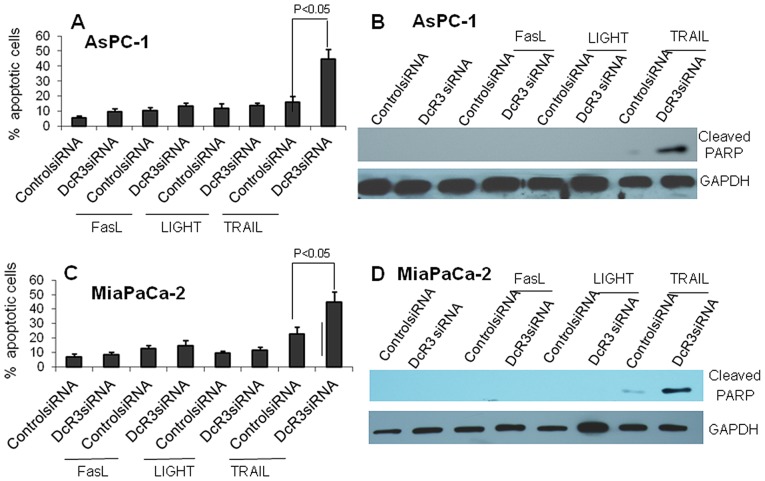
DcR3 siRNA enhanced TRAIL-induced apoptosis. AsPC-1 or MiaPaCa-2 cells were transfected with 10 nM of control siRNA or DcR3 siRNA, respectively. After 24 hours, the cells were treated with recombinant FasL, LIGHT, or TRAIL (100 ng/ml for AsPC-1 and 20 ng/ml for MiaPaCa-2 cells) for 24 hours. Cells were harvested and subjected to flow cytometry for the sub-G1 fraction (**A** and **C**) or Western blotting for cleaved PARP (**B** and **D**). DcR3 siRNA significantly enhanced TRAIL-induced apoptosis (*P*<0.05).

To test if the unmasking of TRAIL could increase its accessibility to its receptor and enhance this death pathway, we examined the effect of DcR3 on TRAIL-mediated apoptosis. The DNA fragmentation (sub-G1) assay showed that when AsPC-1 cells were treated with FasL or LIGHT plus control siRNA or DcR3 siRNA, apoptosis did not increase significantly. However, when treated with TRAIL and DcR3 siRNA, AsPC-1 cells exhibited a dramatic increase in apoptosis, as compared to cells treated with TRAIL plus control siRNA (**Fig 4A**). MiaPACa-2 cells exhibited a similar pattern (**Fig 4C**). The enhanced apoptosis was further confirmed by increased cleaved PARP, as determined by Western blotting (**Fig 4B and 4D**). The results indicate that reduced DcR3 could greatly enhance the proapoptotic effect of TRAIL in these 2 pancreatic cancer cell lines (as compared to FasL and LIGHT), whereas DcR3 is utilized by these cells to counteract the proapoptotic effect of TRAIL.

### DcR3 affected gemcitabine-mediated apoptosis in vitro

**Figure 5:**
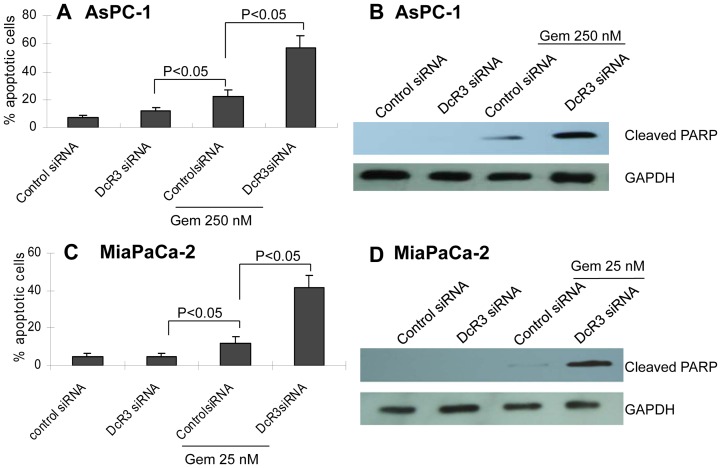
DcR3 siRNA enhanced gemcitabine-induced apoptosis. AsPC-1 or MiaPaCa-2 cells were transfected with 10 nM of control siRNA or DcR3 siRNA, respectively. After 24 hours, the cells were treated with gemcitabine (250 or 25 nM, respectively) for 24 hours. Cells were harvested and subjected to flow cytometry for the sub-G1 fraction (**A** and **C**) or Western blotting for cleaved PARP (**B** and **D**). The combination of DcR3 siRNA and gemcitabine significantly enhanced the proapoptotic effect.

DcR3 siRNA and gemcitabine reduced the expression level of DcR3 (**Fig 3** **and** **4**). We wanted to determine if further downregulation of DcR3 via the combination of these 2 factors could enhance the proapoptotic effect, as compared to a single factor alone. After cells were treated with either siRNA or gemcitabine alone or in combination, DNA fragmentation and cleaved PARP were measured. The results showed that while gemcitabine alone caused increased apoptosis (**Fig 5A and 5C**) and cleaved PARP levels (**Fig 5B and 5D**), its combination with siRNA further increased apoptosis and cleaved PARP levels (**Fig 5**). Without a proapoptotic ligand with which to bind and counteract, DcR3 siRNA alone had no effect; however, when TRAIL was upregulated by gemcitabine, the reduction of DcR3 decreased the masking of TRAIL, thereby promoting apoptosis.

These results are consistent with those in Figure 3. In conjunction with the results shown in Figures 1 and 4, the data suggest that DcR3 binding to TRAIL reduces TRAIL-induced apoptosis and protects tumor cells, which might account for the resistance of tumors to TRAIL treatment.

### Downregulation of DcR3 enhanced the chemotherapeutic effect on tumor growth in vivo

**Figure 6:**
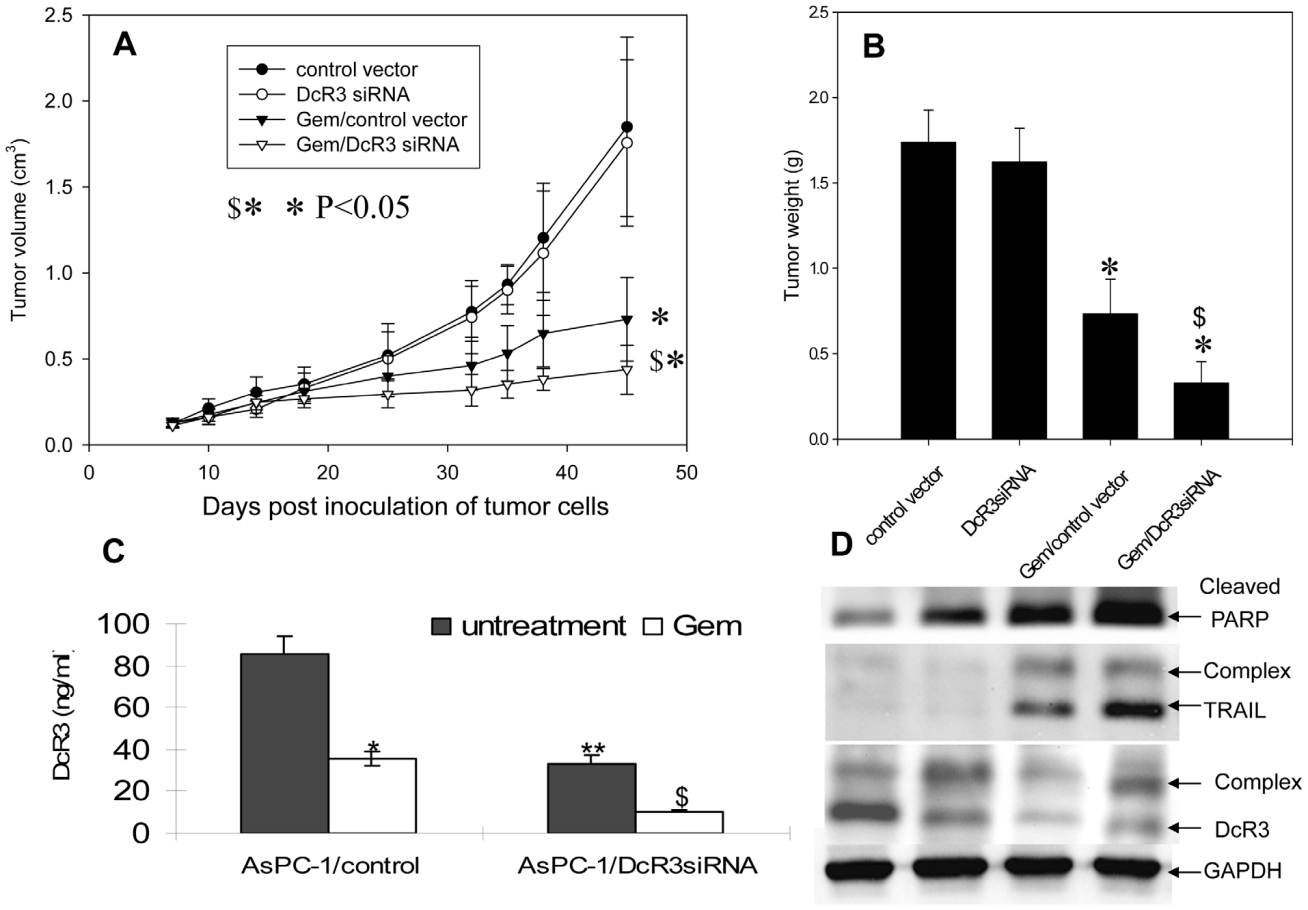
DcR3 siRNA enhanced the antitumor effect of gemcitabine. Control siRNA and DcR3 siRNA-transfected AsPC-1 cells (2×10^6^/site) were subcutaneously injected into nude mice (8 mice/group) and allowed to establish for 7 days. Thereafter, the established tumors were treated with 100 mg/kg of gemcitabine or vehicle (PBS) via intravenous injection twice weekly for 4 weeks. (**A**) The tumor growth curve was calculated from tumor sizes measured twice weekly. The reduction of DcR3 enhanced the antitumor effect of gemcitabine. (**B**) Tumors were weighed at the end of the experiment. * *P*<0.05, tumors in the gemcitabine-treated groups versus in untreated groups in vector-transfected AsPC-1 cells; $* *P*<0.05, tumors in siRNA transfectants versus in vehicle transfectants with gemcitabine treatment. (**C**) The reduction of DcR3 in tumor tissues with different treatments determined with an ELISA. * *P*<0.05, DcR3 in untreated tumors versus in gemcitabine-treated tumors formed by vector-transfected AsPC-1 cells; ** *P*<0.05, DcR3 in tumor control siRNA versus in DcR3 siRNA-transfected AsPC-1 cells; $ *P*<0.05, DcR3 in untreated tumors versus in gemcitabine-treated tumors formed by DcR3 siRNA-transfected AsPC-1 cells. (**D**) Western blotting was performed in tumor lysate (30 μg/sample) collected from each group and stained with primary antibodies of anti-TRAIL, anti-DcR3, anti-cleaved PARP, and anti-GAPDH (as loading control).

Encouraged by promising *in vitro* results, we wanted to determine if the reduction of DcR3 could enhance the effectiveness of chemotherapy *in vivo.* AsPC-1 cells were transfected with a mammalian expression vector that contained siRNA to knockdown the expression of DcR3 and a cassette for G418 selection. The pooled vector mock-transfection cells (as control), or DcR3 siRNA transfected cells with low DcR3 expression (confirmed by ELISA) were injected into nude mice, and the established tumors (60 mm3) were then treated with gemcitabine. The results showed that DcR3 siRNA alone did not slow tumor growth (**Fig 6A**) or reduce tumor weight (**Fig 6B**); however, DcR3 siRNA enhanced the inhibitory effect of gemcitabine, which is related with the enhanced downregulation of DcR3 in the tumor tissue as measured by an ELISA (**Fig 6C** **)**.

In addition, the levels of DcR3 and TRAIL and their complex in the lysate of tumor tissues were examined by Western blotting. Similar to the results found in *in vitro* cell culture lysate (**Fig 1I and 1J**), anti-DcR3 and anti-TRAIL staining resulted in 2 bands (**Fig 6D**), indicating that the DcR3-TRAIL complex exists in tumors. The level of DcR3 was reduced by siRNA, and the level of TRAIL was increased in the gemcitabine-treated group (with or without siRNA), as compared to the vehicle alone or siRNA alone, indicating that gemcitabine upregulates TRAIL and that the downregulation of DcR3 unmasks TRAIL (allowing anti-TRAIL to access TRAIL as evidenced by a strong band in lane 4 of **Fig 6D**). Consistent with the *in vitro* data, TRAIL-induced apoptosis *in vivo* was evidenced by increased cleaved PARP (**Fig 3** **,** **4** **, and** **5**).

There was no difference in tumor growth between the vector-alone mock transfectants and the DcR3 siRNA-transfected cells, suggesting that the existence of proapoptotic molecules (induced by chemotherapeutic agents) is a prerequisite for the action of DcR3. The dual action of gemcitabine, the induction of TRAIL, and the downregulation of its antagonist DcR3 reveal a new mechanism by which gemcitabine exerts its powerful antipancreatic cancer effect.

## Discussion

Pancreatic cancer is a devastating and particularly malignant cancer with a very low 14% 5-year survival rate for stage 1A and an abysmal 1% for stage IV [25]. We speculate that this poor prognosis might be associated with pancreatic cancer's strong ability to resist apoptosis. Using Biacore binding analysis, ELISA, flow cytometry, immunoprecipitation, and Western blotting, we demonstrated that DcR3 binds to TRAIL and decreases TRAIL-mediated apoptosis (**Fig 1**). The reduction of DcR3 unmasks TRAIL on the cell surface and promotes apoptotic signaling of pancreatic cancer cells both *in vitro* and *in vivo*.

Based on the finding that transformed cells and normal cells possess the DR4/DR5 receptors for TRAIL, several studies have determined that TRAIL is a promising molecule for controlling cancer progression [6], [12], [20], [26], [27], [28]. However, because of its antagonist decoy receptor, TRID, which is expressed in normal cells and blocks the apoptotic signaling of DR4/DR5 but is not well expressed in cancer cells [29], [30], [31], the apoptotic signaling of TRAIL is decreased in normal cells but not in tumor cells [23], [24], [32], [33], While the TRAIL-related therapeutic approach has been shown to control the growth of certain types of cancer [31], [34], it has been ineffective for pancreatic cancer [35]. Moreover, its underlying mechanism is largely unknown.

Our study reveals that DcR3 is an antagonist decoy receptor that confers pancreatic cancer cells with resistance to proapoptotic TRAIL. The overexpression of DcR3 has been observed in malignant tumor types, such as pancreatic and ovarian cancer [22], [36]. These highly malignant cells express a higher level of DcR3, as compared to other tumor cells with low malignancy [22], [36], [37]. For example, STS26T, a malignant peripheral nerve sheath tumor cell line that forms aggressive tumors in nude mice, expresses a high level of DcR3, while the T265 and ST88-14 cells that do not form tumors under the same conditions express a low level of DcR3 (our unpublished data). Functionally, the level of DcR3 seems to correlate with resistant to the chemotherapy. To achieve an apoptotic effect similar to that of MiaPACa-2 cells, AsPC-1 cells required 10-fold the amount of gemcitabine. These results demonstrate that AsPC-1 cells with a higher level of DcR3 are more resistant to gemcitabine than MisPACa-2 cells with a lower level of DcR3 (**Fig 3**). The knockdown of DcR3 level enhanced gemcitabine-induced apoptosis both *in vitro* and *in vivo* (**Fig 4** **,** **5** and **6**), which is related to an increased level of TRAIL on the cell surface (**Fig 1B** and **1C**). In contrast, the addition of DcR3 reduced gemcitabine-induced apoptosis (**Fig 3**) via reduction of functionally active TRAIL on the cell surface (**Fig 1B**–**1D**). DcR3 extracellularly binds to TRAIL, thereby masking it and blocking the interaction between TRAIL on the surface of one cell with DR4/DR5 on another.

The binding of DcR3 and TRAIL was seen not only in the setting with 2 pure recombinant proteins but also in the culture media and the lysate of AsPC-1 and MiaPACa-2 cells (**Fig 1G-1L**). The exogenous addition of recombinant DcR3 enhanced the masking of TRAIL on the cell surface, while the reduction of DcR3 unmasked TRAIL, leading to its easy access (**Fig 1B** and **1C**). Furthermore, the enhanced interaction of TRAIL and its receptor led to enhanced activation of apoptosis, as evidenced by increased cleaved PARP and apoptotic cells (**Fig 3** **,** **4** **,** **5**, and **6**). These results indicate that the physical binding of DcR3 to TRAIL confers an antiapoptotic effect.

Although FasL, LIGHT, and TRAIL interacted with DcR3, the reduction of DcR3 with siRNA did not seem to affect FasL-mediated and LIGHT-mediated apoptosis in AsPC-1 and MiaPACa-2 cells. This reduction did dramatically increase TRAIL-mediated cleavage of PARP and the number of apoptotic cells (**Fig 4**), suggesting that TRAIL exerts a dominate apoptotic effect and is sensitive to the masking effect of DcR3 in these 2 pancreatic cancer cell lines.

Although the primary action of gemcitabine is to interrupt cellular DNA synthesis, it also indirectly promotes apoptosis. This study indicates that the underlying mechanism by which gemcitabine induces apoptosis is related to its ability to reduce DcR3 and to increase TRAIL expression (**Fig 2B, 2C**, and **6D**), which allows more TRAIL exposed on the cell surface to exert its proapoptotic function. The reduction of DcR3 with siRNA resulted in a further increase in exposed TRAIL (**Fig 2B** and **C**), which translated into the enhancement of TRAIL-induced apoptosis (**Fig 4** **)**. The combination of gemcitabine with siRNA strongly enhanced apoptosis; this was observed not only *in vitro* (**Fig 4** **and** **5**) but also *in vivo,* as evidenced by the slow growth and lower weight of primary tumors (**Fig 6**). All data indicate that the combination of gemcitabine and DcR3 siRNA reduce the masking of TRAIL and dramatically enhance the drug's therapeutic effect. It is possible that a lower dose of gemcitabine would enhance the effect of the combined treatment, while reducing side effects. Further studies are warranted to examine this possibility.

Notably, our binding data are inconsistent with the finds of Piiti *et al* [22]. TRAIL is most homologous to FasL, sharing 28% of the amino acid sequence identity in their extracellular domains. Their study proved that DcR3 binds to FasL, whereas the TRAIL-Fc overexpression in human 293 cells did not exhibit the same binding [22]. The inconsistence of our observation with the Pitti report might be due to a difference in our assay systems. It remains to be studied whether the machinery required for the formation of the DcR3-TRAIL complex is fully possessed by AsPC-1 pancreatic cancer cells but is missing in TRAIL-Fc overexpressing 293 cells, which might account for the different binding phenotypes. Based on the results of crystal structure analysis and homolog comparisons, the death receptors of type I cells (e.g., DcR3, DR3, DR5, and TNFR) and the death ligands (e.g., FasL, LIGHT, TL1A, and TRAIL) do share functionally similar domains [13]; therefore, it is likely that DcR3 could bind to FasL, LIGHT, TL1A, and TRAIL with different levels of affinity.

The function of DcR3 varies with the cell lines. For example, DcR3 induces apoptosis of dendritic cells, but it protects cancer cells and inversely correlates with the overall survival time of pancreatic cancer patients [38], which may be related to weakening the immune response to tumors. The signaling pathway for the expression of DcR3 might also vary with different cells. Chen *et al* reported that the expression of DcR3 in AsPC-1 cells is related to phosphatidylinositol 3-kinase-, Akt-, and NF-kappaB-dependent pathways [39], which are upregulated in AsPC-1.

Taken together, our data strongly support the notion that tumor cells utilize binding of DcR3 to TRAIL to escape TRAIL-mediated apoptosis, an extracellular protection system for malignancy. As the reduction of DcR3 is likely to tip the balance from antiapoptosis to proapoptosis, it represents a new potential approach to treat pancreatic cancer.

## References

[1] Stauffer SR (2007) Small molecule inhibition of the Bcl-X(L)-BH3 protein-protein interaction: proof-of-concept of an in vivo chemopotentiator ABT-737. Curr Top Med Chem 7: 961–965. 17508927 10.2174/156802607780906843

[2] Catz SD, Johnson JL (2003) BCL-2 in prostate cancer: a minireview. Apoptosis 8: 29–37. 12510149 10.1023/a:1021692801278

[3] Flaherty KT, Stevenson JP, O'Dwyer PJ (2001) Antisense therapeutics: lessons from early clinical trials. Curr Opin Oncol 13: 499–505. 11673691 10.1097/00001622-200111000-00013

[4] Tsujimoto Y (1998) Role of Bcl-2 family proteins in apoptosis: apoptosomes or mitochondria? Genes Cells 3: 697–707. 9990505 10.1046/j.1365-2443.1998.00223.x

[5] Bratton SB, Cohen GM (2001) Apoptotic death sensor: an organelle's alter ego? Trends Pharmacol Sci 22: 306–315. 11395159 10.1016/s0165-6147(00)01718-1

[6] Karikari CA, Roy I, Tryggestad E, Feldmann G, Pinilla C, et al (2007) Targeting the apoptotic machinery in pancreatic cancers using small-molecule antagonists of the X-linked inhibitor of apoptosis protein. Mol Cancer Ther 6: 957–966. 17339366 10.1158/1535-7163.MCT-06-0634 PMC3062431

[7] Cosman D (1994) A family of ligands for the TNF receptor superfamily. Stem Cells 12: 440–455. 7528588 10.1002/stem.5530120501

[8] Ashkenazi A, Dixit VM (1999) Apoptosis control by death and decoy receptors. Curr Opin Cell Biol 11: 255–260. 10209153 10.1016/s0955-0674(99)80034-9

[9] Wallach D, Varfolomeev EE, Malinin NL, Goltsev YV, Kovalenko AV, et al (1999) Tumor necrosis factor receptor and Fas signaling mechanisms. Annu Rev Immunol 17: 331–367. 10358762 10.1146/annurev.immunol.17.1.331

[10] She QB, Bode AM, Ma WY, Chen NY, Dong Z (2001) Resveratrol-induced activation of p53 and apoptosis is mediated by extracellular-signal-regulated protein kinases and p38 kinase. Cancer Res 61: 1604–1610. 11245472

[11] Farrelly N, Lee YJ, Oliver J, Dive C, Streuli CH (1999) Extracellular matrix regulates apoptosis in mammary epithelium through a control on insulin signaling. J Cell Biol 144: 1337–1348. 10087274 10.1083/jcb.144.6.1337 PMC2150575

[12] Ibrahim SM, Ringel J, Schmidt C, Ringel B, Muller P, et al (2001) Pancreatic adenocarcinoma cell lines show variable susceptibility to TRAIL-mediated cell death. Pancreas 23: 72–79. 11451151 10.1097/00006676-200107000-00011

[13] Zhan C, Patskovsky Y, Yan Q, Li Z, Ramagopal U, et al (2011) Decoy strategies: the structure of TL1A:DcR3 complex. Structure 19: 162–171. 21300286 10.1016/j.str.2010.12.004 PMC3065972

[14] Connolly K, Cho YH, Duan R, Fikes J, Gregorio T, et al (2001) In vivo inhibition of Fas ligand-mediated killing by TR6, a Fas ligand decoy receptor. J Pharmacol Exp Ther 298: 25–33. 11408521

[15] Migone TS, Zhang J, Luo X, Zhuang L, Chen C, et al (2002) TL1A is a TNF-like ligand for DR3 and TR6/DcR3 and functions as a T cell costimulator. Immunity 16: 479–492. 11911831 10.1016/s1074-7613(02)00283-2

[16] Yu KY, Kwon B, Ni J, Zhai Y, Ebner R, et al (1999) A newly identified member of tumor necrosis factor receptor superfamily (TR6) suppresses LIGHT-mediated apoptosis. J Biol Chem 274: 13733–13736. 10318773 10.1074/jbc.274.20.13733

[17] Zhang J, Salcedo TW, Wan X, Ullrich S, Hu B, et al (2001) Modulation of T-cell responses to alloantigens by TR6/DcR3. J Clin Invest 107: 1459–1468. 11390428 10.1172/JCI12159 PMC209323

[18] Shi G, Mao J, Yu G, Zhang J, Wu J (2005) Tumor vaccine based on cell surface expression of DcR3/TR6. J Immunol 174: 4727–4735. 15814697 10.4049/jimmunol.174.8.4727

[19] Shrader M, Pino MS, Lashinger L, Bar-Eli M, Adam L, et al (2007) Gefitinib reverses TRAIL resistance in human bladder cancer cell lines via inhibition of AKT-mediated X-linked inhibitor of apoptosis protein expression. Cancer Res 67: 1430–1435. 17308080 10.1158/0008-5472.CAN-06-1224

[20] Xu ZW, Kleeff J, Friess H, Buchler MW, Solioz M (2003) Synergistic cytotoxic effect of TRAIL and gemcitabine in pancreatic cancer cells. Anticancer Res 23: 251–258. 12680221

[21] Chen J, Zhang L, Kim S (2004) Quantification and detection of DcR3, a decoy receptor in TNFR family. J Immunol Methods 285: 63–70. 14871535 10.1016/j.jim.2003.11.004

[22] Pitti RM, Marsters SA, Lawrence DA, Roy M, Kischkel FC, et al (1998) Genomic amplification of a decoy receptor for Fas ligand in lung and colon cancer. Nature 396: 699–703. 9872321 10.1038/25387

[23] Mori T, Doi R, Toyoda E, Koizumi M, Ito D, et al (2005) Regulation of the resistance to TRAIL-induced apoptosis as a new strategy for pancreatic cancer. Surgery 138: 71–77. 16003319 10.1016/j.surg.2005.03.001

[24] Song JJ, An JY, Kwon YT, Lee YJ (2007) Evidence for two modes of development of acquired tumor necrosis factor-related apoptosis-inducing ligand resistance. Involvement of Bcl-xL. J Biol Chem 282: 319–328. 17110373 10.1074/jbc.M608065200

[25] American Cancer Society (2013) Pancreatic Cancer Overview. Available: http://www.cancer.org/acs/groups/cid/documents/webcontent/003071-pdf.pdf. Accessed 14 May 2013.

[26] Hylander BL, Pitoniak R, Penetrante RB, Gibbs JF, Oktay D, et al (2005) The anti-tumor effect of Apo2L/TRAIL on patient pancreatic adenocarcinomas grown as xenografts in SCID mice. J Transl Med 3: 22. 15943879 10.1186/1479-5876-3-22 PMC1156958

[27] Jacob D, Davis JJ, Zhang L, Zhu H, Teraishi F, et al (2005) Suppression of pancreatic tumor growth in the liver by systemic administration of the TRAIL gene driven by the hTERT promoter. Cancer Gene Ther 12: 109–115. 15486557 10.1038/sj.cgt.7700773

[28] Hall MA, Cleveland JL (2007) Clearing the TRAIL for Cancer Therapy. Cancer Cell 12: 4–6. 17613431 10.1016/j.ccr.2007.06.011

[29] Pan G, O'Rourke K, Chinnaiyan AM, Gentz R, Ebner R, et al (1997) The receptor for the cytotoxic ligand TRAIL. Science 276: 111–113. 9082980 10.1126/science.276.5309.111

[30] Pan G, Ni J, Wei YF, Yu G, Gentz R, et al (1997) An antagonist decoy receptor and a death domain-containing receptor for TRAIL. Science 277: 815–818. 9242610 10.1126/science.277.5327.815

[31] Boehrer S, Nowak D, Hoelzer D, Mitrou PS, Chow KU (2006) The molecular biology of TRAIL-mediated signaling and its potential therapeutic exploitation in hematopoietic malignancies. Curr Med Chem 13: 2091–2100. 16918339 10.2174/092986706777935294

[32] Mori T, Doi R, Kida A, Nagai K, Kami K, et al (2007) Effect of the XIAP inhibitor Embelin on TRAIL-induced apoptosis of pancreatic cancer cells. J Surg Res 142: 281–286. 17640673 10.1016/j.jss.2007.03.068

[33] Okamoto K, Ocker M, Neureiter D, Dietze O, Zopf S, et al (2007) bcl-2-specific siRNAs restore gemcitabine sensitivity in human pancreatic cancer cells. J Cell Mol Med 11: 349–361. 17378914 10.1111/j.1582-4934.2007.00013.x PMC3822833

[34] Finnberg N, El-Deiry WS (2006) Selective TRAIL-induced apoptosis in dysplastic neoplasia of the colon may lead to new neoadjuvant or adjuvant therapies. Clin Cancer Res 12: 4132–4136. 16857782 10.1158/1078-0432.CCR-06-0567

[35] Tsuji S, Hosotani R, Yonehara S, Masui T, Tulachan SS, et al (2003) Endogenous decoy receptor 3 blocks the growth inhibition signals mediated by Fas ligand in human pancreatic adenocarcinoma. Int J Cancer 106: 17–25. 12794752 10.1002/ijc.11170

[36] Bai C, Connolly B, Metzker ML, Hilliard CA, Liu X, et al (2000) Overexpression of M68/DcR3 in human gastrointestinal tract tumors independent of gene amplification and its location in a four-gene cluster. Proc Natl Acad Sci U S A 97: 1230–1235. 10655513 10.1073/pnas.97.3.1230 PMC15578

[37] Li H, Zhang L, Lou H, Ding I, Kim S, et al (2005) Overexpression of decoy receptor 3 in precancerous lesions and adenocarcinoma of the esophagus. Am J Clin Pathol 124: 282–287. 16040301 10.1309/XK59-4E4B-5WU8-2QR6

[38] Chang YC, Chen TC, Lee CT, Yang CY, Wang HW, et al (2008) Epigenetic control of MHC class II expression in tumor-associated macrophages by decoy receptor 3. Blood 111: 5054–5063. 18349319 10.1182/blood-2007-12-130609

[39] Chen PH, Yang CR (2008) Decoy receptor 3 expression in AsPC-1 human pancreatic adenocarcinoma cells via the phosphatidylinositol 3-kinase-, Akt-, and NF-kappa B-dependent pathway. J Immunol 181: 8441–8449. 19050262 10.4049/jimmunol.181.12.8441

